# Supplementary material for: Transcriptome Analysis Reveals Vimentin-Induced Disruption of Cell–Cell Associations Augments Breast Cancer Cell Migration
Source: Cells. 2022 Dec 13;11(24):4035. doi: 10.3390/cells11244035 (PMC9776984; doi:10.3390/cells11244035)
Supplement: Supplementary file 1 [file cells-11-04035-s001.zip › DEGs and enrichment analysis.zip!/DEGs and enrichment analysis/WT_VIMvsVector GO enrichment/GSEA/heat_map_corr_plot.html]

Heat map and correlation plot for fpkm  

Fig 1: heat\_map      
 Heat Map of the top 50 features for each phenotype in fpkm

  
  

Fig 2: Ranked Gene List Correlation Profile      
 Ranked list correlations for fpkm

  
  
    
